# Dendritic Cell-Derived Extracellular Vesicles Mediate Inflammation in Egg Allergy Patients

**DOI:** 10.3390/ijms27021042

**Published:** 2026-01-21

**Authors:** Davis Tucis, Georgina Hopkins, Victoria James, David Onion, Lucy C. Fairclough

**Affiliations:** 1 School of Life Sciences, The University of Nottingham, Nottingham NG7 2UH, UK; davis.tucis@nottingham.ac.uk (D.T.); georgina.hopkins@nottingham.ac.uk (G.H.); david.onion@nottingham.ac.uk (D.O.); 2 School of Veterinary Medicine and Science, The University of Nottingham, Nottingham NG7 2UH, UK; victoria.james@nottingham.ac.uk

**Keywords:** type I hypersensitivity, allergy, dendritic cells, extracellular vesicles, T cells, Th2 cells

## Abstract

Atopic allergy is rising globally and placing a significant strain on healthcare systems, yet the understanding of the underpinning mechanisms of allergic sensitization remains incomplete. Extracellular vesicles (EVs) have recently emerged as important mediators of immune modulation, due to their diverse cargo, and therefore may play a mechanistic role in allergic sensitization development. Thus, this study investigated whether EVs released by activated dendritic cells (DCs) contribute to allergic sensitization of the common egg allergen, ovalbumin (OVA). DCs were generated from human monocytes cultured with GM-CSF and IL-4, then stimulated with LPS and/or OVA. EVs were subsequently isolated using size-exclusion chromatography and added to freshly isolated naive T cells at defined time points. T cell responses were then analyzed using spectral flow cytometry. The results highlight that EVs derived from LPS or LPS + OVA-stimulated DCs enhanced IL-4 production and reduced IFN-γ production in naive T cells from egg-allergic donors, indicating a shift toward a Th2 profile. In healthy donors, LPS-induced DC EVs also suppressed IFN-γ expression. Notably, EVs alone were insufficient to activate T cells without CD3/CD28 co-stimulation, suggesting that EVs may function as a “third signal” shaping T cell polarization. These findings highlight a potential role for DC-derived EVs in initiating allergic sensitization.

## 1. Introduction

Allergic diseases driven by IgE-mediated type-I hypersensitivity have become a major global health challenge, with food allergy prevalence reaching 8–10% in children and 3–6% in adults in Westernized countries [1,2]. For egg allergy, it most commonly presents in infancy with a prevalence of about 2% in children and 0.1% in adults [3].

Allergic sensitization is the first stage in developing an allergy. The critical step in allergic sensitization is the aberrant activation of naive CD4^+^ T cells by professional antigen-presenting cells, particularly dendritic cells (DCs), resulting in Th2 polarization characterized by IL-4, IL-5, and IL-13 secretion, IgE class-switching, and subsequent binding to mast cells and basophils [4,5]. Naive T cell priming classically requires three signals: T cell receptor recognition of peptide–MHC-II complexes (signal 1), co-stimulation (primarily CD80/CD86–CD28) (signal 2), and polarizing cytokines that dictate lineage commitment (signal 3) [6].

However, intercellular communication in immunity extends well beyond direct cell–cell contact and cytokines. Extracellular vesicles (EVs), a heterogeneous family of lipid-enclosed particles ranging from 30 to 1000 nm, are now established as important carriers of bioactive cargo, including proteins, lipids, mRNAs, miRNAs, and intact MHC–peptide complexes [7,8,9]. Dendritic cells constitutively release EVs, and their biogenesis, cargo loading, and surface composition are profoundly modulated by maturation status and environmental stimuli, such as pathogen-associated molecular patterns (e.g., lipopolysaccharide, LPS) or allergens [10,11,12]. Numerous murine and human studies have demonstrated that DC-derived EVs can transfer allergen or MHC–allergen complexes to T cells and promote Th2-skewed responses. For instance, EVs from DCs can carry major cat allergen Fel d 1 and induce allergic immune response [1,13]. These findings have led to the hypothesis that EVs may function as an autonomous “signal 3” or amplify classical priming pathways during allergic sensitization [14].

Despite substantial progress, several methodological limitations have prevented definitive conclusions regarding the specific contribution of human DC-derived EVs to allergy. Most functional studies have relied on differential ultracentrifugation, which co-isolates soluble proteins, immune complexes, and lipoproteins that independently affect T cell responses [15,16]. Residual cytokines frequently co-purified with EVs can mask vesicle-specific effects. Many assays have employed total or memory CD4^+^ T cells rather than rigorously purified naive populations, making it difficult to distinguish genuine sensitization from recall responses. Furthermore, prolonged DC culture in serum-containing media introduces substantial bovine EV contamination, breaking current MISEV guidelines [7,17]. Finally, direct comparative studies between healthy individuals and patients with confirmed allergy using cytokine-depleted, highly purified EVs remain scarce.

To address these challenges, the present study established a fully human, MISEV-compliant in vitro platform that incorporates optimized monocyte-derived DC differentiation with short-term (24 h) maturation in serum-free medium, EV isolation by size-exclusion chromatography with validated depletion of soluble cytokines, comprehensive multimodal EV characterization, and repeated exposure of stringently isolated naive CD4^+^ T cells from healthy donors and individuals with egg allergy, both with and without CD3/CD28 co-stimulation. This work aimed to determine whether highly purified human DC-derived EVs generated under resting, inflammatory (LPS), allergen (OVA), or combined conditions can activate and polarize naive CD4^+^ T cells, and whether their immunomodulatory effects differ between healthy and allergic individuals, thereby clarifying the role of EVs in the early events of human allergic sensitization.

## 2. Results

### 2.1. Generation of Dendritic Cells in Serum-Free Conditions for EV Analysis

**Figure 1:**
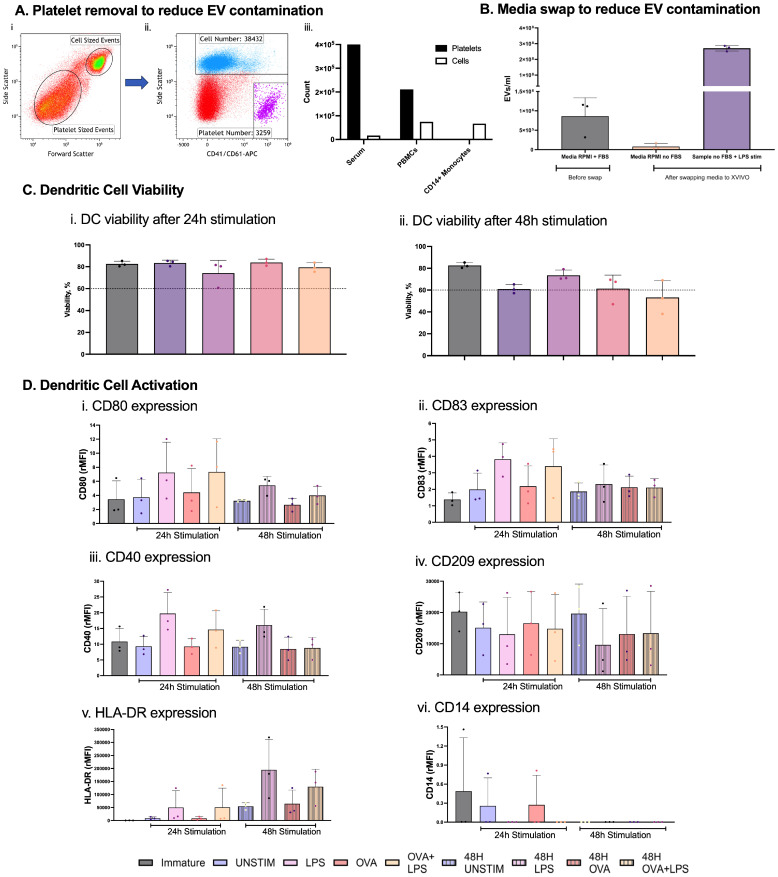
**Extracellular vesicles (EVs) and dendritic cell (DC) optimization.** Platelet removal analysis following CD14+ magnetic isolation (**Ai**–**iii**) and complete media swap from RPMI + 10% FBS to serum-free X-VIVO 15 on day 5, immediately before DC stimulation, as well as EV measurement after stimulation (**B**). Viability assessed by Zombie Aqua (**C**) and maturation marker expression (rMFI) on DCs (**D**) after 24 h and 48 h stimulation. *n* = 3 independent healthy donors.

To ensure purity in EV isolates for assessing allergic sensitization, strategies were implemented to deplete platelet-derived EVs and soluble cytokines, which could confound T cell responses. Platelet contamination in serum-supplemented media was examined following CD14+ monocyte isolation (Figure 1(Ai)). Forward scatter analysis of CD41/CD61 events revealed a marked reduction in platelet counts post-isolation of CD14+ magnetic isolation (Figure 1(Aii,iii)). Due to the requirement of culturing monocytes in media containing serum to maintain viability, EV carryover from the monocyte-to-DC culture was mitigated by swapping RPMI + 10% FBS to serum-free X-VIVO prior to stimulation (Figure 1B). ImageStreamX analysis showed a ~70% decrease in baseline EV concentration post-swap. Furthermore, when compared to LPS-stimulated DC production of EVs, media EV presence accounted for less than 1% of total EVs produced by DCs.

Monocyte-derived DCs were then stimulated with 100 ng/mL LPS, 10 µg/mL OVA, or OVA + LPS for 24 or 48 h to assess both viability (Figure 1(Ci,ii)) and maturation marker expression (Figure 1D) of the DCs. Viability, assessed by Zombie Aqua staining, exceeded 70% at 24 h across conditions, meeting MISEV guidelines, but declined slightly at 48 h. Maturation was also evaluated via flow cytometry for costimulatory (CD80, CD83, and CD40) and adhesion/activation (CD209, HLA-DR, and CD14) markers. At 24 h, LPS and OVA + LPS stimulation upregulated CD80, CD83, and CD40 relative to immature/unstimulated DCs, indicative of full maturation (Figure 1D). OVA alone elicited minimal changes. At 48 h, expression waned, mirroring viability trends. CD209 decreased selectively in LPS-stimulated DCs, while HLA-DR increased. CD14 remained low across conditions (Figure 1D). These data confirm that 24 h stimulation with LPS or OVA + LPS in X-VIVO yields viable, mature DCs with minimal contaminants from serum-based and platelet-derived EVs, optimizing EV production for T cell studies. As expected, there is some donor variation in the data, but donor origin did not affect either viability or maturation.

Concerning cytokine removal, molecular weight filters are often used for concentrating EVs after isolation, but their ability to remove cytokines from a sample is yet to be tested. EVs are often concentrated from larger pools of media before SEC. We tested for the presence of cytokines and total EVs pre- and post-concentration with molecular weight cut-off filters and size-exclusion chromatography. As activated and mature human monocyte-derived DCs produce copious amounts of IL-6 and IL-8, the presence of these cytokines was measured in the EV fractions after SEC, and after Amicon filter column centrifugation (Supplementary Figure S1).

The results show that both IL-6 and IL-8 were still present in the top chamber of filters where EVs were also present, in both 10 kDa and 100 kDa filters (Supplementary Figure S1i), suggesting that these are not suitable for EV/cytokine separation. Furthermore, analysis of EVs showed that both the 10 and 100 kDa columns lost EVs after centrifugation. In contrast, there were no cytokines detected in SEC fractions one to nine (Supplementary Figure S1ii), while maintaining the majority of EVs, with most EVs present in fractions 1–5. For all future experiments, the generated samples were isolated by SEC and fractions 1–7 were combined to ensure that EVs can be added to naive T cells without any contaminating cytokine.

### 2.2. Characterization of EVs Generated by DCs

**Figure 2:**
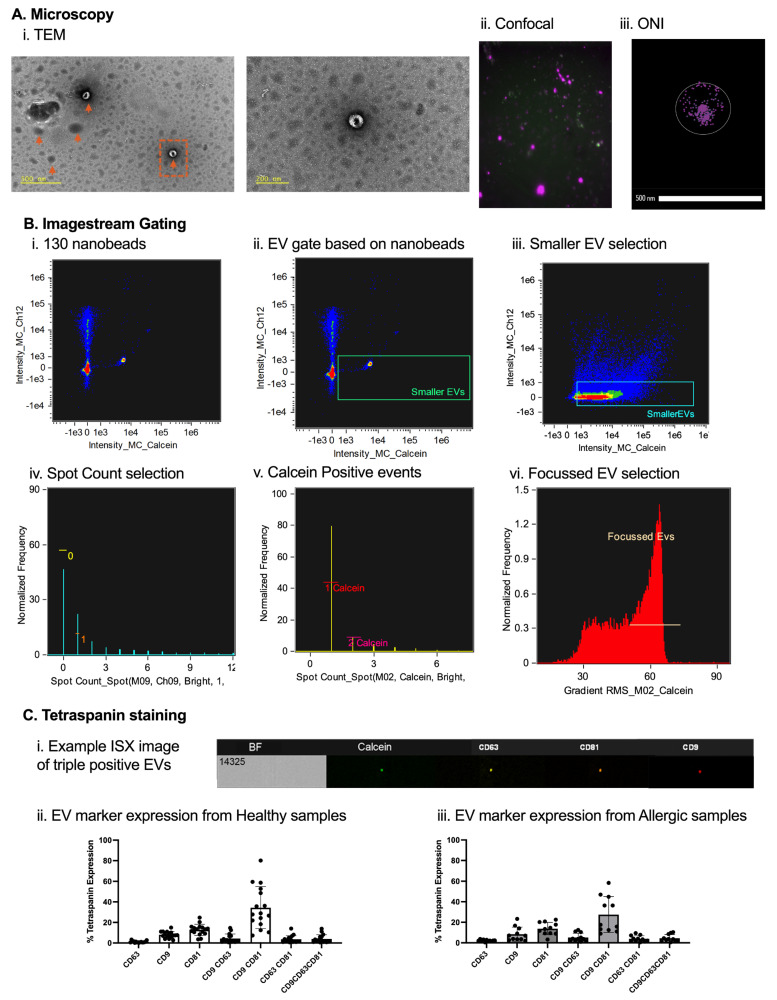
**Extracellular vesicle (EV) analysis.** DC-derived EVs were observed under transmission electron microscopy (TEM) (**Ai**). Utilizing confocal microscopy, calcein-AM-stained and lipid dye DID-stained EVs were examined (**Aii**). Super-resolution microscope (ONI) allowed the staining of tetraspanins CD81, CD63, and CD9 to observe the number of copies on each EV in the sample (**Aiii**). Nanobeads of known refractive index and sized at 130 nm were run on Imagestream (ISX) to determine their positioning on the machine (**Bi**). Based on the interest in small EVs, everything below 130 nm beads was selected to include all biological EVs below 300 nm in size (**Bii**). The sizing gate was then applied to EV samples (**Biii**). Intensity_MC_12 is Side Scatter. ISX brightfield’s minimum pixel range is 0.9 µm; a gate ensuring objects larger than 0.9 µm were excluded was also set (**Biv**). To ensure objects selected were intact EVs, a calcein+ gate was introduced (**Bv**). To reduce artefacts and increase the accuracy of tetraspanin expression on EVs, only in-focus EVs were selected (**Bvi**). Normalized frequency represents the relative proportion of events. ImageStream X MkII imaging of a single EV showing the EV brightfield image (BF), calcein, CD63 PE, CD81 PE-Vio 615, and CD9 APC fluorescence (**Ci**), and tetraspanin expression on DC-derived EVs from healthy (**Cii**) and allergic individuals (**Ciii**). *n* = 6 healthy and *n* = 5 allergic participants.

EV preparations from monocyte-derived DCs were characterized using multimodal imaging and flow-based analysis to confirm morphology, size, and molecular markers. Transmission electron microscopy (TEM) revealed characteristic cup-shaped, collapsed vesicles with diameters of 50–150 nm (Figure 2(Ai)). Confocal microscopy of calcein-AM (green)- and lipid dye DiD (red)-stained EVs displayed punctate, colocalized signals consistent with intact lipid bilayers (Figure 2(Aii)). Super-resolution nanoscopy (ONI) identified tetraspanin distribution, with CD81, CD63, and CD9 exhibiting clustered expression on vesicle surfaces (Figure 2(Aiii)). For high-throughput analysis, EVs were also evaluated on imaging flow cytometry using 130 nm calibration nanobeads to establish size thresholds; as it was not possible to use Mie Theory on ISX, 130 nm beads were used as a reference point for EV size selection. It is well known that nanobeads appear higher on SSC than EVs because of their reflective index, estimating that 130 nm nanobeads equate roughly 300 nm for biological EVs (Figure 2(Bi)). Events below this threshold were gated as EVs (Figure 2(Bii)), with further refinement to exclude particles >1 µm via brightfield area (Figure 2(Biii)). Single calcein-AM-positive vesicles were isolated using spot count masking (Figure 2(Biv)), including only calcein-positive events (Figure 2(Bv)), yielding a focused EV population with modal intensity ~60 (Figure 2(Bvi)). Tetraspanin expression was then profiled on calcein-gated EVs from healthy and egg-allergic donor DCs using a REAfinity antibody panel (Figure 2C). EVs predominantly expressed CD9 and CD81 in both donor types, with lower CD63 levels. Co-expression analysis revealed frequent single (CD9/CD81) and double (CD9/CD81) positivity, alongside triple-positive subpopulations. These characterisations validate the purity and identity of DC-derived EVs for downstream functional assays.

### 2.3. Minimal Impact of DC-Derived EVs on Naive T Cell Viability, Activation, and Cytokine Production

**Figure 3:**
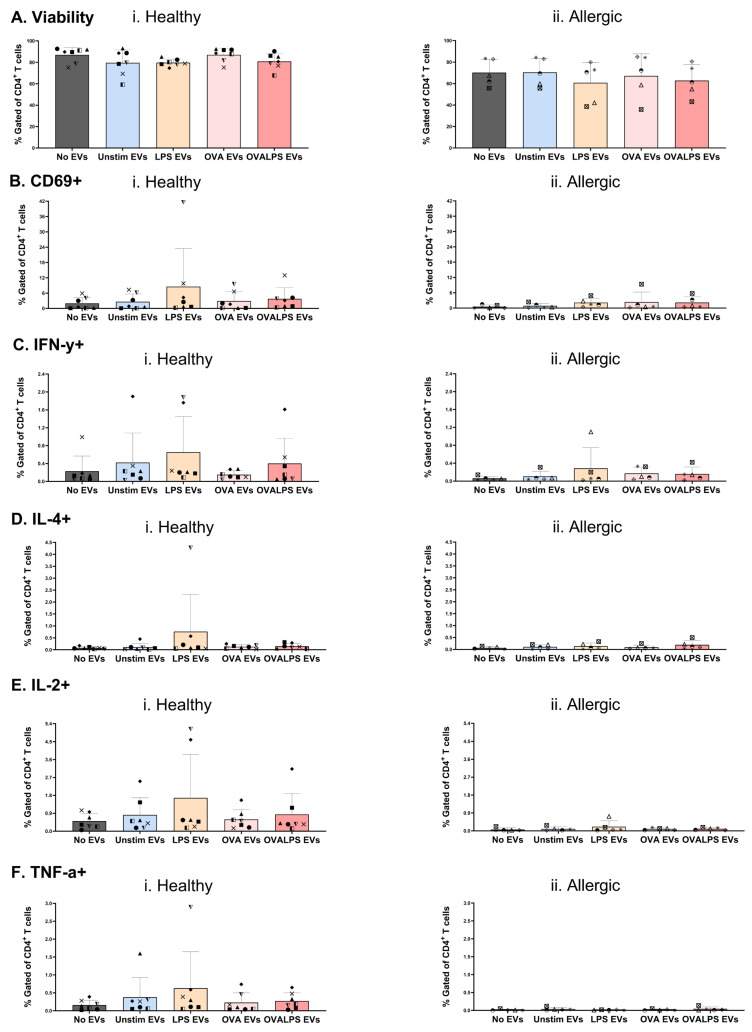
**Effect of extracellular vesicles (EVs) alone on T cell viability and function.** DCs were stimulated with 100 ng/mL of LPS, 10 µg/mL of OVA, 50 ng/mL of LPS, and 10 µg/mL of OVA, and unstimulated; PBS was added as control. T cells were stimulated every 24 h for 3 doses of dendritic cell-derived EVs; only 3rd dose results are shown. T cell viability was first assessed (**Ai**,**ii**). Cytokine expression was also measured after 42 h, following 30 h stimulation of EVs and 12 h incubation with protein transport inhibitor (PTI), CD69 (**Bi**,**ii**), IFN-y (**Ci**,**ii**), IL-4 (**Di**,**ii**), IL-2 (**Ei**,**ii**), and TNF-a (**Fi**,**ii**). *n* = 6 healthy and *n* = 5 allergic participants, each identified by a different symbol. Statistical analysis was performed in GraphPad Prism Version 10.6.1 using one-way ANOVA, *p* < 0.05.

Naive T cells from healthy donors and allergic individuals were exposed three times to EVs derived from unstimulated, LPS-stimulated, OVA-stimulated, or OVA + LPS-stimulated DCs. The viability of the T cells remained high across all conditions and donor types (Figure 3A). The activation of T cells, as identified by early activation marker CD69 expression, was negligible in both groups (Figure 3B), indicating limited T cell priming by EVs alone. Furthermore, intracellular cytokine staining revealed minimal Th1/Th2/Th17-associated responses following EV exposure (namely, IFN-γ [Figure 3C], IL-4 [Figure 3D], TNF-α [Figure 3E], and IL-2 [Figure 3F]). These data demonstrate that DC-derived EVs alone elicit no stimulation of naive T cells, regardless of the DC stimulation state or donor allergy status.

### 2.4. Enhanced T Cell Activation and Th2 Skewing with CD3/CD28 Co-Stimulation

**Figure 4:**
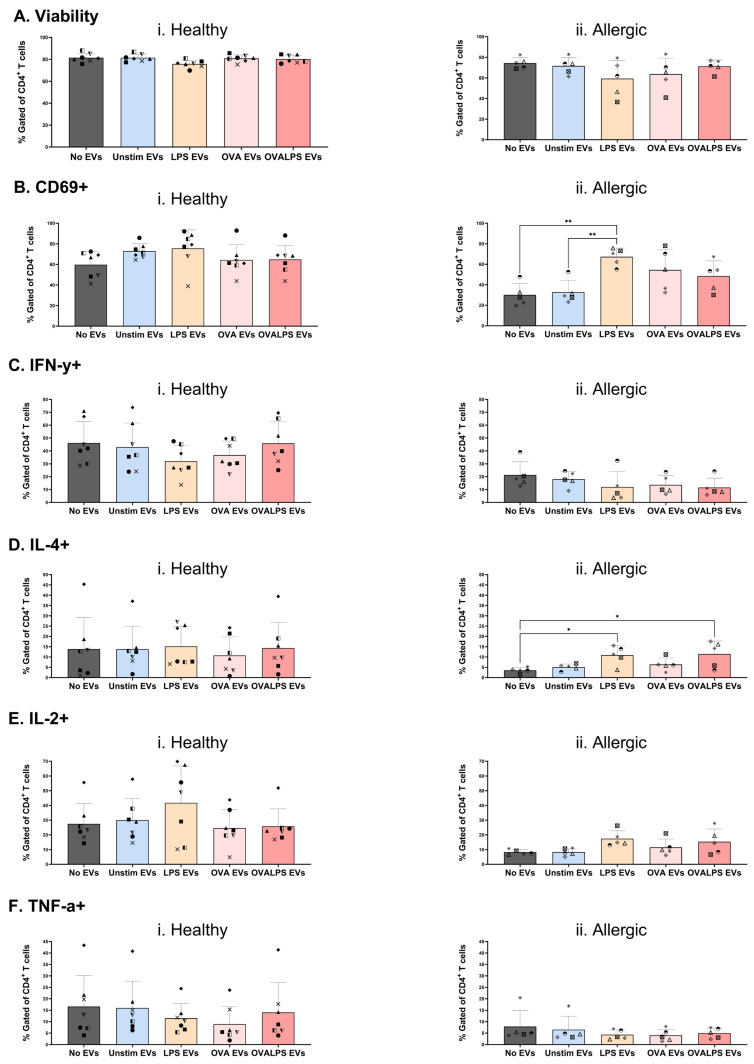
**Extracellular vesicles (EVs) as signal 3 in T cell activation.** Naive T cells were plated in 96-well plate with co-stimulation of coated 2 µg/mL CD3 and direct 2 µg/mL CD28. Each well was then stimulated with dendritic cell-derived EVs added in 3 doses. DCs were stimulated with 100 ng/mL of LPS, 10 µg/mL of OVA, 50 ng/mL of LPS, and 10 µg/mL of OVA, and unstimulated; PBS was added as control. T cells were stimulated every 24 h 3 times. T cell viability was first assessed (**Ai**,**ii**). Cytokine expression was measured after 42 h, following 30 h stimulation of EVs and 12 h incubation with protein transport inhibitor (PTI), CD69 (**Bi**,**ii**), IFN-y (**Ci**,**ii**), IL-4 (**Di**,**ii**), IL-2 (**Ei**,**ii**), and TNF-a (**Fi**,**ii**). *n* = 6 healthy and *n* = 5 allergic participants, each identified by a different symbol. Statistical analysis was performed in GraphPad Prism Version 10.6.1 using one-way ANOVA, *p* < 0.05.

To evaluate the synergistic effects of DC-derived EVs with concomitant T cell receptor signalling, naive T cells from healthy and allergic donors also underwent three sequential exposures to EVs (generated from unstimulated, LPS-stimulated, OVA-stimulated, or OVA + LPS-stimulated DCs) in the presence of anti-CD3/CD28 co-stimulation. This co-stimulation amplified overall T cell responses compared to EV exposure alone. Specifically, T cell viability was largely preserved across conditions (Figure 4A), exceeding 80% in healthy donors and ~70% in allergic donors. CD69 was expressed in both donor types after 96 h culture (Figure 4B); however, T cells from allergic patients displayed significantly lowered activation in the absence of EVs or when EVs were derived from unstimulated DCs, when compared to EVs derived from either LPS- or LPS/OVA-stimulated DCs (Figure 4(Bii)). Overall, the percentage of IFN-γ-producing T cells (Th1 marker) was higher in T cells from healthy donors (20–30% positive; Figure 4(Ci)) and comparatively subdued in T cells from allergic donors (10–20%; Figure 4(Cii)), but this was irrespective of the presence or absence of EVs. Conversely, the percentage of IL-4-producing T cells (Th2 marker) in healthy samples was found at modest levels (Figure 4(Di)), as was the case for allergic T cells (Figure 4(Dii)); however, in allergic individuals, the addition of EVs generated from either LPS- or LPS + OVA-stimulated DCs significantly increased the percentage of IL-4-positive T cells. The percentage of IL-2-positive T cells, supporting T cell expansion, was comparable in both groups (Figure 4E), as was the percentage of TNF-α-positive T cells (pro-inflammatory marker), and again this was irrespective of the presence or absence of EVs. These results illustrate that CD3/CD28 co-stimulation unmasks EV-driven T cell activation, with allergic donors showing biased type 2 responses that could drive allergic disease progression.

### 2.5. Unsupervised Clustering Analysis Reveals Differences in Baseline T Cell Responses Generated in Healthy and Allergic Individuals

**Figure 5:**
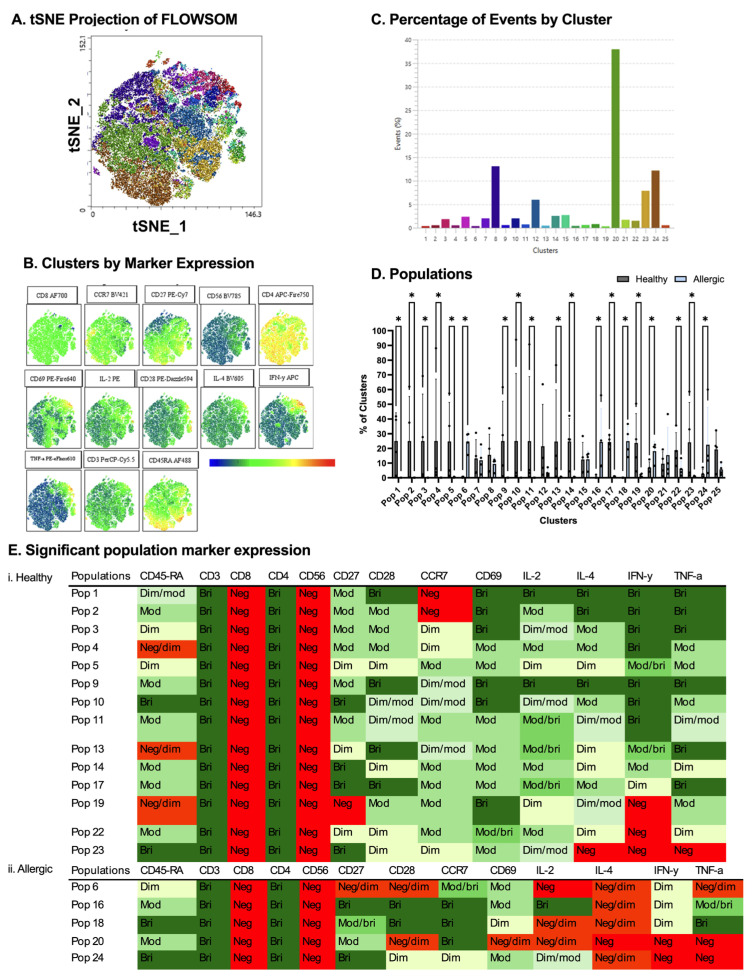
**Comparison between healthy and allergic groups using unbiased clustering analysis following culture with PBS-stimulated dendritic cell-derived extracellular vesicles (EVs).** Following cluster explorer plugin, a multi-coloured tNSNE plot was made (**A**) and individual marker clusters were produced from tSNE plot (**B**). Clusters were separated based on their percentage (**C**). Populations between healthy and allergic groups were analyzed for significant differences using Mann–Whitney tests *p* < 0.05 (**D**). Significant populations were split between healthy (**Ei**) and allergic groups (**Eii**), and their marker expression was colour coded—Negative (red), Negative/dim (orange), DIM (pale yellow), Dim/moderate (pale green), Moderate (light green), Moderate/bright (green), and Bright (dark green) (**Ei**,**ii**). *n* = 4 healthy donors and *n* = 4 allergic donors. * *p* < 0.05.

To further delineate the effects of EVs generated from PBS-stimulated DCs (i.e., baseline effects) on naive T cell subsets between healthy and allergic donors, flow cytometry data from viable CD3+CD4+ T cells from four healthy and four allergic donors were subsampled (5000 events/donor) and subjected to FlowSOM clustering (20 meta-clusters) incorporating surface (CD45RA, CD8, CD56, CD27, CD28, CCR7, and CD69) and intracellular (IL-2, IL-4, IFN-γ, and TNF-α) markers. Figure 5A presents the tSNE visualization of FlowSOM clusters, Figure 5B is the tSNE visualization of marker density, and Figure 5C presents the percentage of T cells found in each identified FlowSOM cluster, with cluster 20 comprising the largest proportion of events. Of the 25 clusters identified, population distributions differed significantly across 20 clusters (Figure 5D; * *p* < 0.05, Mann–Whitney U). Healthy donor T cells enriched clusters 1–5, 9–11, and 17 (Figure 5(Ei)), which were characterized by high CD45RA, CD27, CD28, and CCR7 (naive/memory precursor phenotype) alongside modest IL-2/TNF-α. Conversely, allergic T cells dominated clusters 6, 16, 18, 20, and 24 (Figure 5(Eii)), marked by elevated IL-4, IFN-γ, and TNF-α with reduced CD45RA/CD27.

### 2.6. Unsupervised Clustering Analysis of T Cells Exposed to EVs Derived from LPS-Stimulated DCs Reveals Differences in T Cell Responses Generated in Healthy and Allergic Individuals

**Figure 6:**
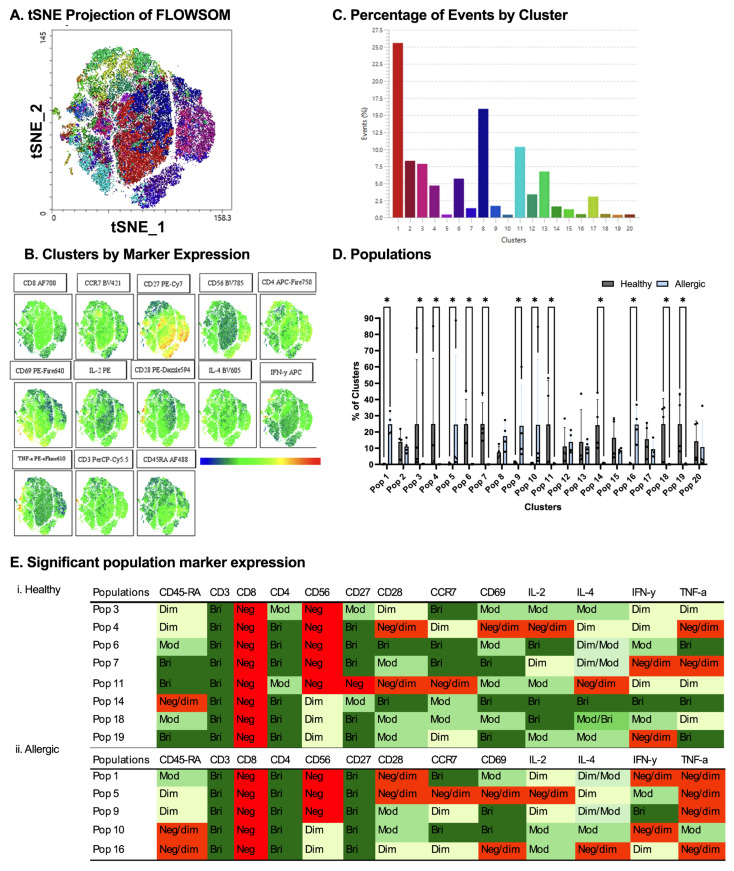
**Comparison between healthy and allergic groups using unbiased clustering analysis following culture with LPS-stimulated dendritic cell-derived extracellular vesicles (EVs).** Following cluster explorer plugin, a multi-coloured tNSNE plot was made (**A**) and individual marker clusters were produced from tSNE plot (**B**). Clusters were separated based on their percentage (**C**). Populations between healthy and allergic groups were analyzed for significant differences using Mann–Whitney tests *p* < 0.05 (**D**). Significant populations were split between healthy (**Ei**) and allergic groups (**Eii**), and their marker expression was colour coded—Negative (red), Negative/dim (orange), Dim (pale yellow), Dim/moderate (pale green), Moderate (light green), Moderate/bright (green), and Bright (dark green) (**Ei**,**ii**). *n* = 4 healthy donors and *n* = 4 allergic donors. * *p* < 0.05.

Having identified differences in both the percentage of CD69-expressing T cells and the percentage of IL-4-producing T cells in the allergic group following the addition of EVs generated from DCs stimulated with LPS, viable CD3+CD4+ naive T cells from four healthy and four allergic donors, stimulated with anti-CD3/CD28 plus EVs from LPS-stimulated DCs, were subsampled (5000 events/donor) and clustered via FlowSOM. A total of 20 meta-clusters were identified using surface (CD45RA, CD8, CD56, CD27, CD28, CCR7, and CD69) and intracellular (IL-2, IL-4, IFN-γ, and TNF-α) markers were also identified. Figure 6A presents the tSNE visualization of FlowSOM clusters, Figure 6B is the tSNE visualization of marker density, and Figure 6C presents the percentage of T cells found in each identified FlowSOM cluster, with cluster 1 dominating. Thirteen clusters exhibited significant population disparities (* *p* < 0.05, Mann–Whitney U; Figure 6D). Healthy T cells preferentially populated clusters 3, 4, 6, 7, 11, 14, and 18 (Figure 6(Ei)), defined by high CD45RA, CD27, CCR7, and IL-2/TNF-α (naive/effector precursor traits). Allergic T cells enriched clusters 1, 5, 9, and 16 (Figure 6(Eii)), particularly, cluster 1 was dominant (CD45RA+, CD3+, CD4+, CD27+, CCR7+, and CD69 low/-), marked by minimal activation and subdued cytokine expression (IL-2/IL-4/IFN-γ/TNF-α low). This LPS-EV exposure preserved donor-specific divergences, with allergic enrichment in minimally activated subsets implying selective Th2 priming or anergy induction by inflammatory EVs in sensitized individuals.

No clustering differences were observed following repeated exposure to EVs from unstimulated, OVA-stimulated, or OVA + LPS-stimulated DCs (see Supplementary Figures S2–S4).

## 3. Discussion

Allergic diseases are a growing global health burden, with rising prevalence and incomplete understanding of the mechanisms underlying allergic sensitization—the aberrant Th2-biased response to harmless antigens. In recent years, EVs have emerged as crucial mediators of intercellular communication and immune modulation, attracting significant interest for their potential roles in allergy pathogenesis and progression [18,19]. EVs are lipid bilayer-enclosed nanoparticles released by diverse cell types, including immune cells (e.g., dendritic cells, mast cells, and T cells), epithelial cells, and non-host sources such as pollen or microbes. They transport bioactive cargo—proteins, lipids, mRNAs, and miRNAs—enabling functional molecule transfer to recipient cells [14,20].

The present study establishes a rigorous, MISEV-compliant human in vitro platform to evaluate the immunomodulatory effects of dendritic cell-derived extracellular vesicles (DC-EVs) on naive CD4^+^ T cells polarization in the context of egg allergy. Our findings indicate that DC-EVs isolated by size-exclusion chromatography (SEC) with cytokine depletion showed minimal impact on T cell viability, CD69 expression, or cytokine secretion in the absence of TCR signalling (both signal 1 and 2). This observation underscores the limitations of earlier studies using ultracentrifugation, which often co-isolates soluble factors capable of independently driving T cell responses [21,22]. In addition, the use of SEC-purified EVs aligns with recent reports emphasizing the need for contaminant-free preparations to accurately evaluate vesicle-specific functions [23].

However, when the EVs are combined with signal 1 and 2 co-stimulation (i.e., CD3/CD28), EVs from LPS- and OVA + LPS-stimulated DCs promote activation and a shift toward type 2 immunity cytokine production, particularly IL-4, which is more pronounced in allergic donors. This allergen-specific type 2 immunity bias was accompanied by reduced IFN-γ (see Supplementary Figure S5), suggesting a role for DC-EVs in amplifying Th2 polarization during sensitization.

These results significantly add to prior work on DC-EVs in allergy. For instance, Fang et al. showed that plasma EVs from allergic rhinitis patients exhibit antigen-presenting properties and drive Th2 differentiation [24], while Molfetta et al. demonstrated that mast cell-derived EVs amplify allergic inflammation via immune complex transfer [25]. In murine models, OVA-loaded mesenchymal stem cell EVs, in contrast, have shown immunosuppression and prevention of allergic sensitization and inflammation [26,27]. In addition, EVs contribute to allergen transport, antigen presentation, Th2 polarization, and inflammation amplification. Dendritic cell-derived EVs shuttle MHC-peptide complexes and co-stimulatory molecules, promoting type 2-skewed responses [1,13]. Epithelial- and mast cell-derived EVs facilitate barrier penetration and effector phase amplification, while pollen-derived “pollensomes” and indoor dust act as stable allergen carriers enhancing immunogenicity [28,29].

High-dimensional FlowSOM analysis further highlighted baseline differences in naive T cell subsets between healthy and allergic donors, with T cells from allergic donors showing subtle enrichment in low-activation clusters. OVA-EV exposure eliminated these differences, while LPS-EVs preserved donor-specific patterns. The enhanced IL-4 response to OVA-EVs in allergic donors suggests that DC-EVs may sustain Th2 memory in established allergies, contributing to persistence beyond infancy. This aligns with evidence that allergen-bearing EVs can facilitate remote antigen presentation, offering opportunities for EV-based diagnostics or therapies, such as targeted depletion during desensitization [30,31].

While this study provides valuable insights into the potential role of dendritic cell-derived extracellular vesicles (DC-EVs) in promoting Th2 polarization in egg allergy using a rigorous, MISEV-compliant in vitro platform, several limitations should be acknowledged. The study was conducted with a relatively small sample size, including only six healthy donors and five egg-allergic participants for the main functional assays, and even fewer (*n* = 4 per group) for the unsupervised clustering analyses, which reduces statistical power and may limit the generalizability of the observed donor-specific differences in T cell responses. The experiments relied on an artificial in vitro co-culture system using monocyte-derived DCs generated with GM-CSF and IL-4, along with stringently isolated naive CD4^+^ T cells. These monocyte-derived DCs may not fully reflect the phenotype or functional properties of naturally occurring human dendritic cell subsets, such as conventional DCs, potentially introducing biases in EV production and immunomodulatory effects. Ovalbumin was used as the model allergen, which, although well-characterized and convenient, does not represent a clinically relevant natural food allergen in terms of human exposure routes or molecular complexity. It lacks the post-translational modifications and contextual factors found in actual egg allergens, limiting the direct translational relevance to human egg allergy pathogenesis. Additionally, while EV morphology, size, and surface markers, such as tetraspanins, were thoroughly characterized, the study did not conduct an in-depth analysis of the internal cargo, including proteins, miRNAs, or lipids, which would be necessary to uncover the specific mechanistic drivers of type 2 immunity skewing. Intracellular cytokine production in T cells was assessed at a single time point (after approximately 36 h of cumulative exposure, including a final 12 h stimulation block). Given that different cytokines exhibit distinct production kinetics—some peaking early while others increase later—this fixed endpoint measurement may have missed transient or delayed cytokine responses, potentially underestimating dynamic shifts in T cell polarization. These limitations underscore the need for future studies with larger cohorts, more physiological models, comprehensive EV cargo profiling, and multiple time point cytokine assessments to further strengthen the evidence supporting the role of DC-EVs in allergic sensitization.

In summary, our data demonstrate that purified DC-EVs can serve as effective signal 3 modulators in naive T cell priming, with OVA + LPS-matured EVs preferentially inducing IL-4 in the context of egg allergy. These findings also provide a foundation for exploring EVs as biomarkers or intervention targets in allergic sensitization. Future studies should incorporate longitudinal donor sampling and the multi-omics of EV cargo to further delineate mechanisms.

## 4. Materials and Methods

### 4.1. PBMC Isolation

Whole blood (50 mL) was collected from healthy and egg-allergic human volunteers into EDTA-coated tubes (approved by the NHS Health Research Authority Research Ethics Committee (Ref 21/SC/0183)). Egg-allergic patients were identified using NHS records of GP notes stating adverse reactions to egg with positive allergy testing results (using either RAST or Skin Prick Testing). The blood was diluted 1:1 (*v*/*v*) with phosphate-buffered saline (PBS) supplemented with 2% fetal bovine serum (FBS) (Merck, Feltham, UK). SepMate^TM^ tubes (StemCell Technologies, Cambridge, UK) were pre-filled with 15 mL Histopaque-1077 (Merck, Feltham, UK), and the diluted blood was carefully layered atop the density gradient. Tubes were centrifuged at 1200 *g* for 10 min at room temperature with the brake engaged. The peripheral blood mononuclear cell (PBMC)-containing buffy coat layer was aspirated and transferred to a new 50 mL conical tube, then diluted to 50 mL with PBS + 2% FBS. Cells were pelleted by centrifugation at 400 *g* for 8 min at room temperature, and the supernatant was discarded. This washing step was repeated once more to remove residual Histopaque and cellular debris. Isolated PBMCs were resuspended in complete RPMI 1640 medium (supplemented with 10% FBS, 1% penicillin-streptomycin, and 2 mM L-glutamine) (Merck, Feltham, UK).

### 4.2. DC Generation and Culture

CD14+ monocytes were isolated from PBMCs using magnetic bead-based positive selection (Miltenyi Biotec, Bergisch Gladbach, Germany) according to the manufacturer’s protocol. Isolated monocytes were resuspended at 1 × 10^6^ cells/mL in RPMI 1640 medium supplemented with 10% FBS, 1% penicillin-streptomycin, and 2 mM L-glutamine, and plated in 48-well flat-bottom tissue culture-treated plates (Corning, Corning, NY, USA). For differentiation into immature DCs (iDCs), 50 ng/mL recombinant human GM-CSF and 20 ng/mL recombinant human IL-4 (both from R&D Systems, Minneapolis, MN, USA) were added. Cultures were maintained at 37 °C in a humidified 5% CO_2_ incubator for 5 days, with 50% media and cytokine replenishment on day 3. On day 5, non-adherent and loosely adherent cells were gently harvested, and the medium was replaced with serum-free X-VIVO 15 (Lonza, Walkersville, MD, USA). iDCs were then stimulated with 100 ng/mL LPS (from Escherichia coli O111:B4; Sigma-Aldrich, St. Louis, MO, USA) and/or 10 µg/mL ovalbumin (OVA; Grade V, Sigma-Aldrich) for 24 h to induce maturation into mature DCs (mDCs). Both LPS and OVA was added to media-only samples and stained with calcein-AM to ensure there was no EV contamination. Maturation status was confirmed by flow cytometric analysis of surface markers (e.g., CD80, CD83, CD86, and HLA-DR). The cells were removed from the cell culture and centrifuged with 1 mL of phosphate buffer albumin (PBA) at 300 *g* for 5 min; the supernatant was kept for use in EV isolation and the pellet was re-suspending by gentle tapping. Antibodies were then added to the FACS tubes per the manufacturer’s instructions and incubated at 4 °C for 30 min. After incubation, 2 mL of PBA was added and washed at 300 *g* for 5 min. Supernatants were discarded and 300 µL of fixation buffer was added. The samples were analyzed on a SONY ID7000 flow cytometer.

### 4.3. Naive T Cell Isolation

Naive CD4+ T cells were isolated from matched donor whole blood via negative magnetic selection using the Naive CD4+ T Cell Isolation Kit II (Miltenyi Biotec, Bergisch Gladbach, Germany), following the manufacturer’s instructions. PBMCs were first isolated as described above. Isolated PBMCs were resuspended in MACS buffer (PBS containing 2 mM EDTA and 0.5% BSA) at 1 × 10^7^ cells/mL. Naive CD4+ Biotin-Antibody Cocktail II (10 µL per 10^7^ cells) was added, and the mixture was incubated at 4 °C for 5 min. Subsequently, MACS buffer (30 µL per 10^7^ cells) and Naive CD4+ MicroBeads II (20 µL per 10^7^ cells) were added, followed by incubation at 4 °C for 10 min. Labelled cells were loaded onto a pre-equilibrated LS MACS column in the magnetic field (Miltenyi Biotec, Bergisch Gladbach, Germany). Unlabelled naive CD4+ T cells in the flow-through were collected, while the column was washed thrice with 3 mL MACS buffer. The eluted fraction was centrifuged at 300 *g* for 5 min at 4 °C, and the cell pellet was resuspended in serum-free AIM-V medium (Thermo Fisher Scientific, Waltham, MA, USA) at 1 × 10^6^ cells/mL. Naive T cells (2 × 10^5^ cells in 200 µL) were aliquoted into 96-well U-bottom tissue culture plates (Corning) for downstream assays. Purity was verified by flow cytometry (>95% CD3+CD4+CD45RA+CCR7+). Extracellular markers were stained by the previously described method.

### 4.4. EV Isolation with Size-Exclusion Chromatography

Following 24 h stimulation with LPS and OVA, DC culture supernatants were harvested and centrifuged at 300 *g* for 8 min at 4 °C to remove cells. Clarified supernatants were subjected to size-exclusion chromatography (SEC) using qEVoriginal/35 nm columns (IZON Science, Oxford, UK) equilibrated with sterile-filtered PBS (0.22 µm pore size). Columns were pre-washed with 2 mL PBS, excess buffer was removed, and 1 mL of supernatant was loaded per column. As the sample entered the resin bed, an additional 1 mL of PBS was added to initiate elusion. Nine EV-enriched fractions (corresponding to elution volumes 1.5–4.5 mL 500 µL each) were collected based on the manufacturer’s guidelines. Fractions were pooled and concentrated using Amicon Ultra-0.5 10 kDa centrifugal filter units (Merck Millipore, Burlington, MA, USA). Pooled eluates (up to 500 µL 9 fractions for cytokine experiment and 7 fractions for DC-T cell assay) were loaded into filters and centrifuged at 4000 *g* for 10 min at 4 °C. Retentates containing concentrated EVs were recovered from the upper chamber and resuspended in 100–200 µL PBS. The EVs were used immediately for the 1st dose of EVs, and for subsequent use, were stored in −20 °C for 24 h until the next dose.

### 4.5. TEM and Confocal Microscopy

To confirm the presence of DC-generated EVs, samples were stained with either calcein-AM or the lipid dye 1,1′-dioctadecyl-3,3,3′,3′-tetramethylindodicarbocyanine, 4-chlorobenzenesulfonate salt (DID). Calcein-AM was prepared according to the manufacturer’s instructions. Briefly, 50 µL of DMSO was added to 50 µg of calcein-AM, resulting in 500 µg/mL, and was vortexed until calcein-AM was fully dissolved. Cells were removed from the samples by centrifugation, and EVs were isolated utilizing size-exclusion columns collecting particles from 35 nm to 350 nm size. EVs were then fixed in 3% glutaraldehyde solution in cacodylate buffer for 30 min. A total of 10 ul of the sample was added to poly-L-Lysine-treated carbon film slot grids (EM resolutions) and left to settle for 15 min. Samples were then washed twice with ddH20 and stained with 1% uranyl acetate for 5 min; excess was removed using blotting paper. TEM was carried out using a Tecnai Biotwin-12 with an accelerating voltage of 100 kV. In TEM, EVs appeared to be cup-shaped and collapsed due to being dried during the preparation of the sample for TEM. Differentiating EVs from other particles was performed by looking for curved, darker inner circles suggesting that there was a membrane present before it was dried.

### 4.6. Super-Resolution Microscopy

DCs were cultured as above, and stimulated with 100 ng/mL LPS to produce EVs. The EVs were isolated using SEC and concentrated utilizing 10 kDa Amicron concentration columns. To stain EVs for the ONI nanoimager, the ONI EV Profiler 2 kit was used with anti-CD9 AF488, anti-CD63 AF568, and anti-CD81 AF647; these antibodies were diluted in 1:100 in staining buffer. A total of 10 µL of EV solution was mixed with the antibody mixture for 50 min at room temperature with gentle rocking. The excess antibodies were washed by centrifugation. A total of 20 µL of fixation buffer was used to stabilize the labelled EVs for 5 min and they were washed again. It was necessary to prepare an ONI assay chip by applying 1 s of surface reagent to each lane, incubating for 15 min with rocking, and then washing with wash buffer. Then, 10 µL of capture reagent was applied for 15 min. Furthermore, 10 µL of stained EVs was added to the chip and incubated for 75 min, followed by washing unbound EVs with wash buffer, and applying 20 µL of fixation buffer for 10 min. The chip was then ran on an ONI nanoimager S for analysis. The data was analyzed using the company’s software CODI Version 0.19.5.

### 4.7. Enzyme-Linked Immunosorbent Assay

After SEC had been performed, each of the fractions (1 to 7) were collected and used in an ELISA to evaluate the effectiveness of the SEC-clearing capabilities of cytokines from EV solutions. The 96-well plates were coated with IL-6 and IL-8 capture antibodies by adding 100 µL of coating solution in each well, ensuring that the solution covers the bottom of each well evenly. The plate was then incubated overnight at 4 °C to allow the capture antibodies to adsorb on the bottom of the wells. After incubation, the coating solution was discarded, and the wells were washed with PBS + 0.05%Tween20. To prevent non-specific binding of proteins to the plate, the plates were blocked with blocking buffer for 1 h. After the blocking of the plate, the blocking buffer was removed, and the wells were washed with PBS + 0.05%Tween20. The sample was then added to the wells and incubated for 2 h at room temperature to allow the cytokines to bind to the capture antibodies. The wells were then washed with PBS + 0.05%Tween20 to remove unbound substances and minimize background noise. Horseradish peroxidase (HRP) Streptavidin was added to the wells and incubated for 1 h at room temperature. The wells were washed again. 3,3′,5,5′ tetramethylbenzidine (TMB) was added to the wells to initiate a reaction with HRP and the sample was allowed to incubate for 20 min in the dark at room temperature. The stop solution (Sulfuric acid) was added to cease the reactions between HRP and TMB. The plate was then inserted into a microplate reader to measure the absorbance at 450 nm and 570 nm. The results from the standards were then plotted in a standard curve. Utilizing the standard curve, it was possible to calculate the concentrations of IL-6 and IL-8 in the solution.

### 4.8. DC-T Cell Assay

Isolated DC-derived EVs were added to naive CD4+ T cell cultures (2 × 10^5^ cells/well in 200 µL AIM-V medium), with EV replenishment every 24 h for up to 3 days. For co-stimulation assays, 96-well U-bottom plates were pre-coated for 1 h at 4 °C with 2 µg/mL anti-human CD3 (clone OKT3; BioLegend, San Diego, CA, USA) in PBS and were washed twice with PBS. Anti-human CD28 (2 µg/mL; clone CD28.2; BioLegend, San Diego, CA, USA) was added directly to wells. Cultures were incubated at 37 °C in a humidified 5% CO_2_ incubator for 72 h total. After 30 h of stimulation, protein transport inhibitor cocktail (GolgiPlug; BD Biosciences, Franklin Lakes, NJ, USA; 1 µL/mL) was added, and cells were incubated for an additional 12 h. Harvested cells were washed once with PBA (PBS containing 0.1% BSA and 0.09% sodium azide) by centrifugation at 300 *g* for 10 min at 4 °C. For extracellular staining, cells were resuspended in 100 µL PBA containing fluorochrome-conjugated antibodies against CD3, CD4, CD45RA, CD27, CD28, CCR7, and CD69 (all from BioLegend, San Diego, CA, USA) by adding 2 µL of each antibody and were incubated at 4 °C for 30 min in the dark. Cells were washed twice with PBA (300 *g*, 5 min) and fixed in 500 µL fixation buffer (BD Biosciences, Franklin Lakes, NJ, USA) for 30 min at 4 °C. For intracellular staining, fixed cells were washed with 2 mL 1× Perm/wash buffer (BD Biosciences, Franklin Lakes, NJ, USA; 500 *g*, 5 min) and were resuspended in 100 µL of Perm/wash buffer containing antibodies against IL-2, IL-4, IFN-γ, and TNF-α (all from BioLegend, San Diego, CA, USA) by adding 2.5 µL of each antibody. Samples were incubated at room temperature for 30 min in the dark, washed twice with PBA (500 *g*, 5 min), and resuspended in 300 µL of fresh fixation buffer for storage at 4 °C until acquisition. Stained samples were acquired on a Sony ID7000 spectral flow cytometer (Sony Biotechnology, Tokyo, Japan) using 405 nm, 488 nm, 561 nm, and 638 nm lasers, collecting data for 2 min per sample at a flow rate of ~1000 events/s. Spectral unmixing and compensation were performed with Sony software (v2.0.4), followed by manual gating in Kaluza Version 2.1 and FlowJo software Version 10.10.

### 4.9. Supervised Flow Cytometry Analysis

Acquired spectral flow cytometry data were analyzed using Kaluza Analysis software (v2.1; Beckman Coulter, Brea, CA, USA). A manual hierarchical gating strategy was applied: the initial selection of viable singlets (forward/side scatter area vs. height, followed by live/dead amine-reactive dye exclusion), then CD3+CD4+ lymphocytes, and further subsetting to naive T cells (CD45RA+CCR7+). Expression of activation (CD69), costimulatory (CD27, CD28), and chemokine receptor (CCR7) markers, along with intracellular cytokines (IL-2, IL-4, IFN-γ, TNF-α), was quantified as a percentage positive within the naive CD4+ gate. Gating was performed on fluorescence-minus-one controls for compensation and isotype controls for positivity thresholds. Aggregated data were imported into GraphPad Prism (v10.1.1; GraphPad Software, San Diego, CA, USA) for visualization. Statistical significance was assessed using two-way ANOVA with Sidak’s post hoc correction for donor group comparisons (healthy vs. allergic; α = 0.05), and one-way ANOVA with the Brown–Forsythe correction for condition effects within groups (unstimulated vs. EV-stimulated; α = 0.05). Differences were deemed significant at *p* < 0.05.

### 4.10. Unsupervised Flow Cytometry Analysis

Flow cytometry data were additionally processed in FlowJo v10.8.1 (BD Biosciences, Franklin Lakes, NJ, USA) for unsupervised clustering. Following import, samples underwent manual hierarchical gating to select viable CD3+CD4+ naive T cells (CD45RA+CCR7+). The ‘Downsample’ plugin was applied to standardize each sample to 10,000 events, minimizing batch effects. Samples were concatenated by experimental condition (e.g., donor group or EV stimulation type) to generate combined files. The optimal cluster number was estimated using the ‘Phenograph’ plugin. This value was input into the FlowSOM plugin to delineate self-organizing map clusters. Resultant clusters were visualized via tSNE. ‘Cluster Explorer’ was employed to generate annotated t-SNE plots (colour-coded by marker expression or population frequency) and quantify event distributions per cluster.

### 4.11. Statistical Analysis

Statistical analyses were performed using GraphPad Prism version 10.1.1 (GraphPad Software, San Diego, CA, USA). Data are presented as individual data points with median and interquartile range or as mean ± SEM where indicated. Normality was assessed using the Shapiro–Wilk test. For comparisons between healthy and allergic donor groups across multiple conditions, two-way repeated-measures ANOVA was used, followed by Šídák’s multiple comparisons test. Differences between stimulation conditions within the same donor group were evaluated by one-way repeated-measures ANOVA with the Brown–Forsythe correction or Friedman test, followed by Dunn’s multiple comparisons test where appropriate. Differences were considered statistically significant at *p* < 0.05. For statistical comparisons of the unsupervised clustering analysis, the percentage of naive T cells per cluster was calculated for each donor. Data were exported to GraphPad Prism and analyzed using multiple Mann–Whitney U tests (unpaired, two-tailed) with false discovery rate (FDR) correction at 5% via the two-stage step-up (Benjamini, Krieger, and Yekutieli) method for healthy vs. allergic donor differences. Condition-specific effects within allergic donors were evaluated by multiple Wilcoxon matched-pairs signed-rank tests with the same FDR correction (α = 0.05).

**Figure 7:**
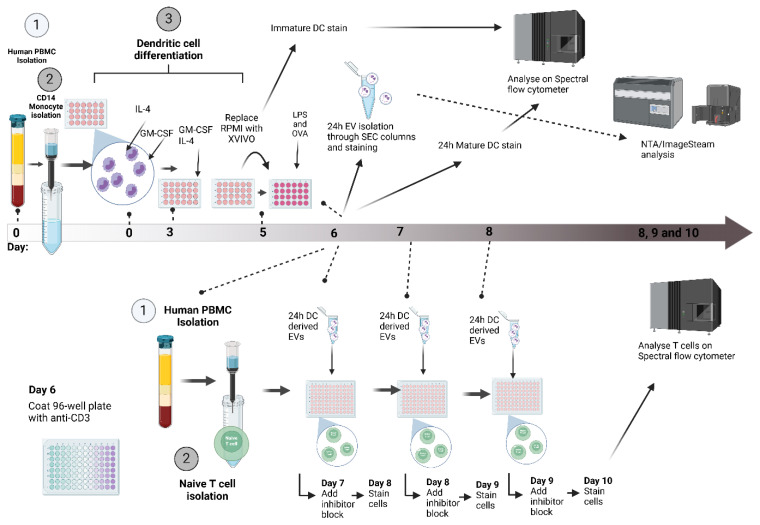
**Dendritic cell-derived extracellular vesicle and T cell assay setup.** On day 0, human whole blood was collected, and PBMCs were isolated by density gradient (1), followed by CD14+ monocyte isolation using magnetic separation (2). Monocytes were cultured in 48-well tissue culture plates with GM-CSF and IL-4 stimulation in RPMI + 10% FBS to initiate differentiation in dendritic cells; on day 3, the media and cytokines were replenished. On day 5, the media was swapped to serum-free media X-VIVO and stimulated with LPS, OVA, and OVA + LPS (3). On day 6, EVs from DCs were collected and isolate through SEC, alongside PBMC 2nd isolation and negative selection of naive T cells using magnetic separation (1,2). Naive T cells were exposed to DC EVs for 3 separate doses, and the cells were collected and stained for extracellular and intracellular markers.

The overall assay workflow is illustrated in Figure 7.

## Data Availability

The original contributions presented in the study are included in the article; further inquiries can be directed to the corresponding author.

## Supplementary Materials

The following supporting information can be downloaded at: https://www.mdpi.com/article/10.3390/ijms27021042/s1.

## Abbreviations

The following abbreviations are used in this manuscript:

BSA	Bovine serum albumin
CD	Cluster of differentiation
DC	Dendritic cell
EDTA	Ethylenediaminetetraacetic acid
EV	Extracellular vesicle
FBS	Fetal bovine serum
IFN	Interferon
IgE	Immunoglobulin E
IL	Interleukin
LPS	Lipopolysaccharide
MHC	Major histocompatibility complex
MISEV	Minimal information for studies of extracellular vesicles
OVA	Ovalbumin
PBS	Phosphate-buffered saline
SEC	Size-exclusion chromatography
t-SNE	t-distributed stochastic neighbour embedding
Th2	T helper 2 cells
TNF	Tumour necrosis factor
