# Supplementary material for: Dendritic Cell-Derived Extracellular Vesicles Mediate Inflammation in Egg Allergy Patients
Source: Int J Mol Sci. 2026 Jan 21;27(2):1042. doi: 10.3390/ijms27021042 (PMC12842409; doi:10.3390/ijms27021042)
Supplement: Supplementary file 1 [file ijms-27-01042-s001.zip › ijms-4067746-supplementary.pdf]

i. Cytokine removal using Amicron columns

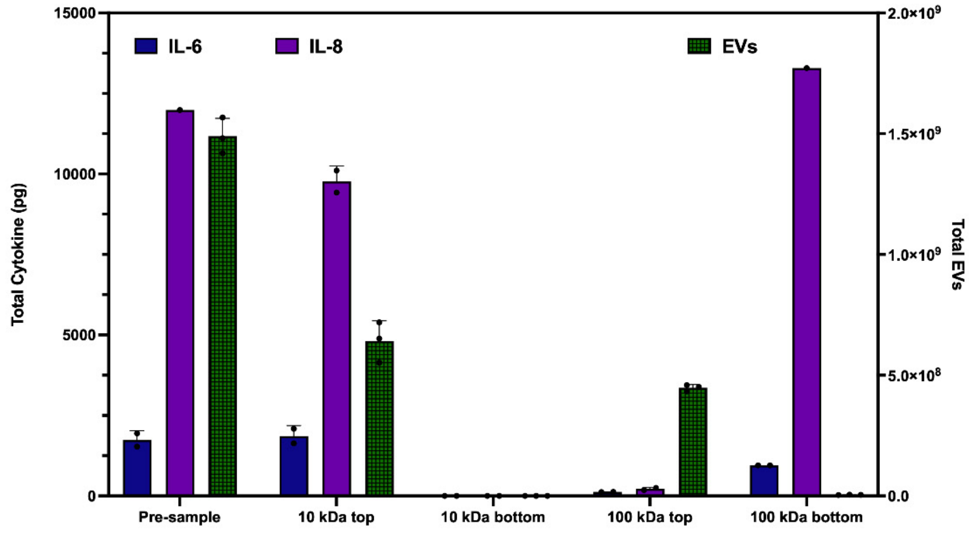

ii. Cytokine removal using SEC

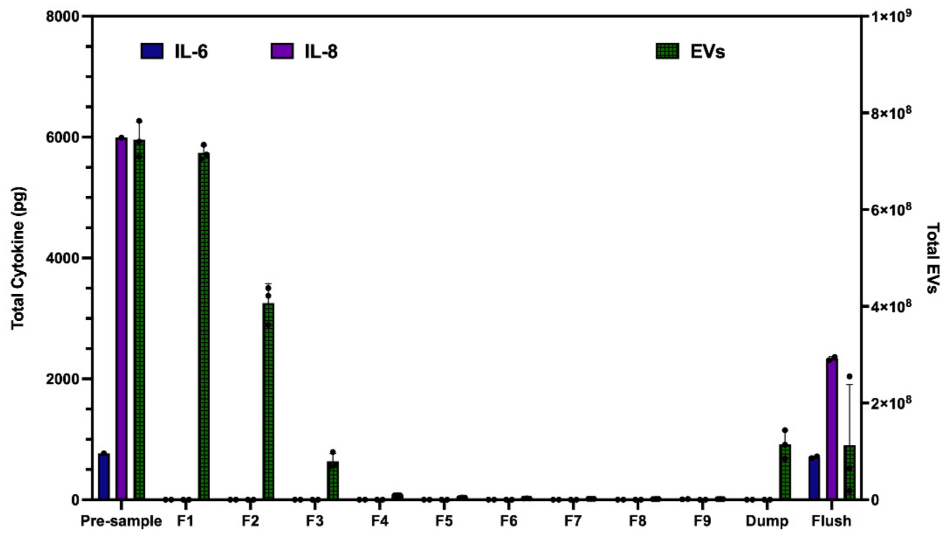

Supplementary Figure S1 - Primary human DC cell supernatants were spiked with recombinant IL-6 and IL-8, processed, and analysed by ELISA (cytokines) and calcein-AM/ImageStreamX (EVs) after Amicon filter column centrifugation (i) or after SEC (ii). n = 3 independent donors.

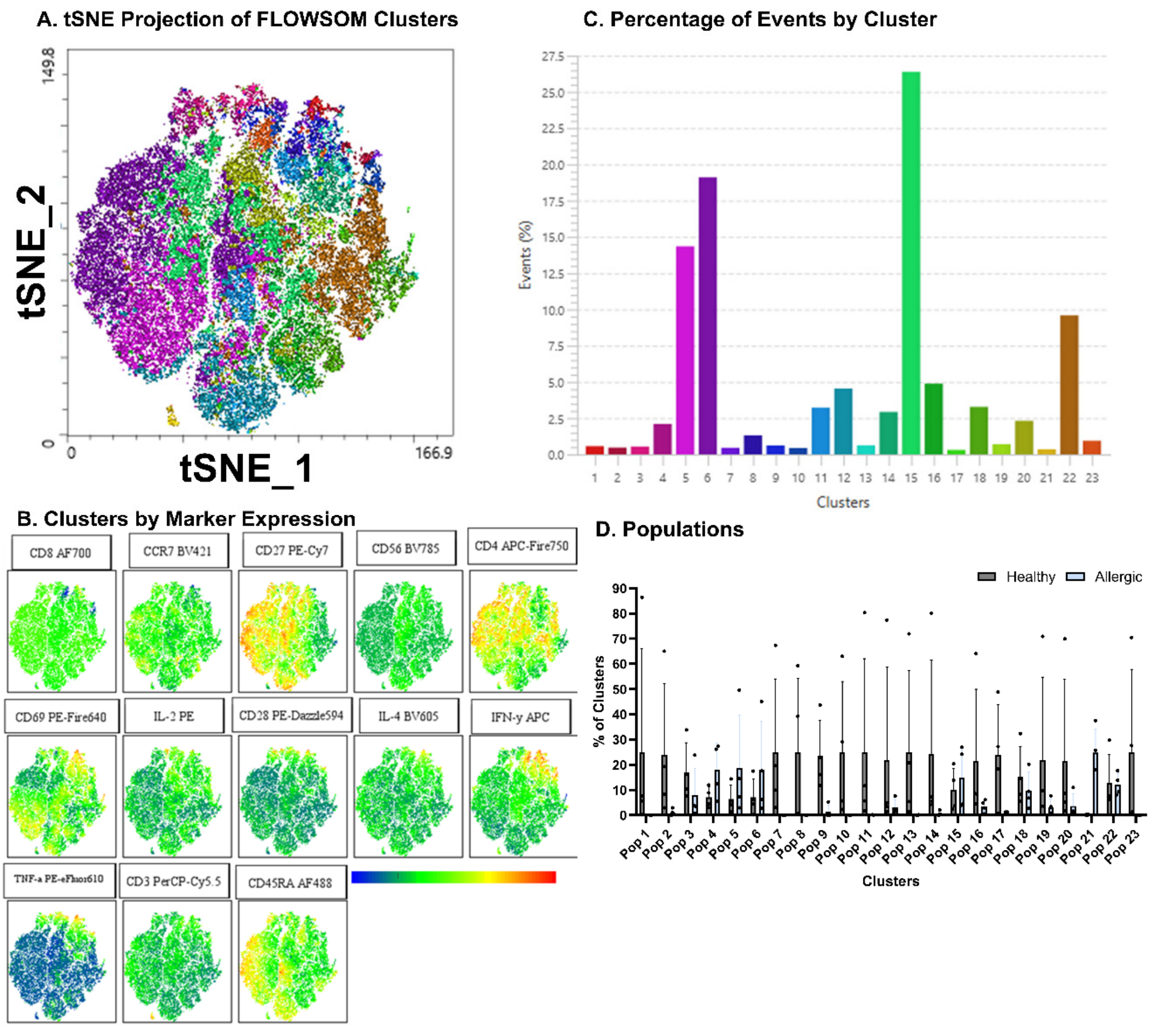

Supplementary Figure S2 - Comparison between healthy and allergic using unbiased clustering analysis following culture with OVA-stimulated DC-derived EVs. Following cluster explorer plugin, a multi-coloured tNSNE plot was made (A) and individual marker clusters were produced from tSNE plot (B). Clusters were separated based on their % (C). (C). Populations between healthy and allergic groups were analysed for significant differences using Mann-Whitney Tests p<0.05 (D). N= 4 healthy donors, 4 allergic donors.

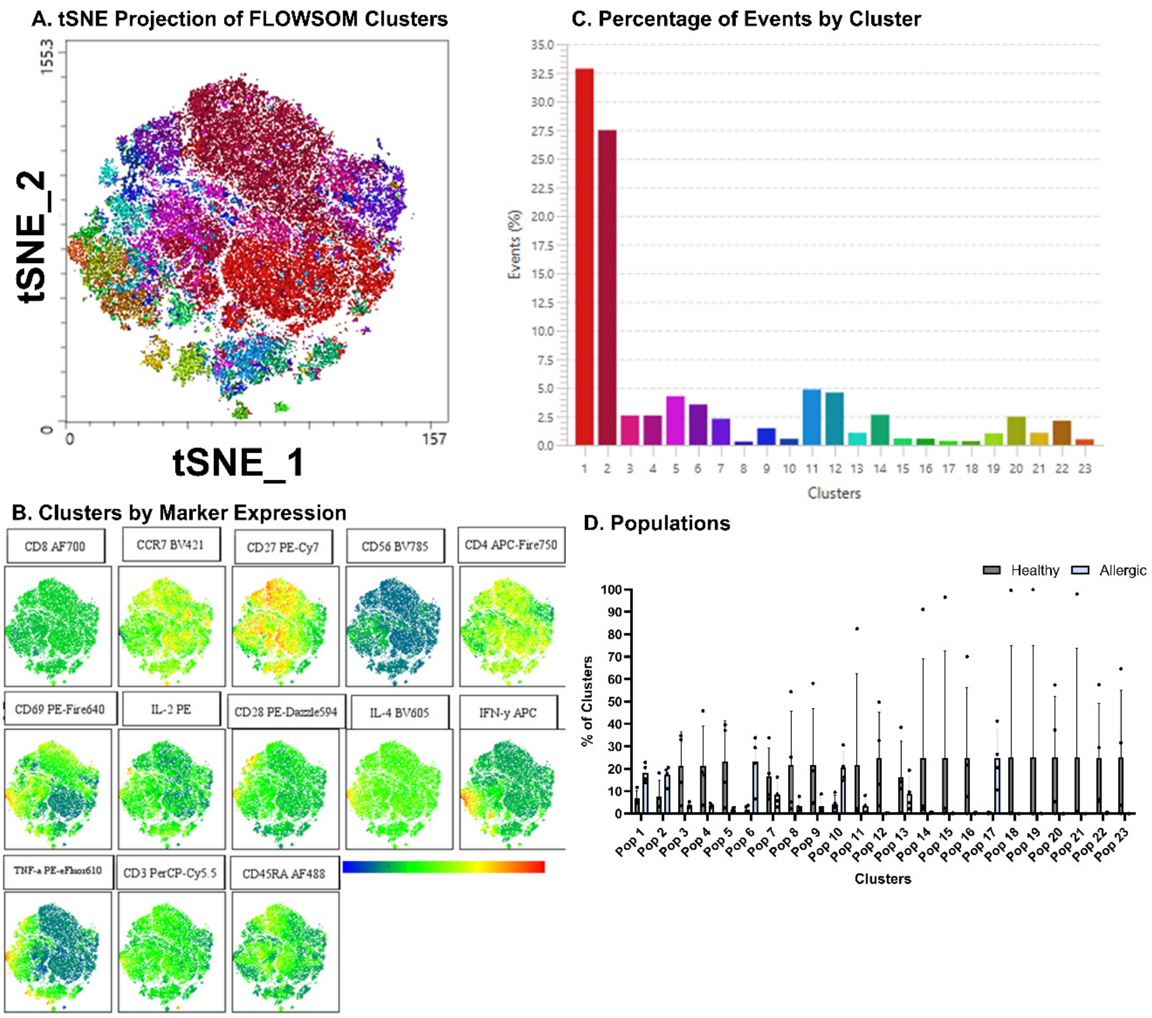

Supplementary Figure S3 - Comparison between healthy and allergic using unbiased clustering analysis following culture with OVA+LPS-stimulated DC-derived EVs. Following cluster explorer plugin, a multi-coloured tNSNE plot was made (A) and individual marker clusters were produced from tSNE plot (B). Clusters were separated based on their % (C). (C). Populations between healthy and allergic groups were analysed for significant differences using Mann-Whitney Tests $p<0.05$ (D). N= 4 healthy donors, 4 allergic donors.

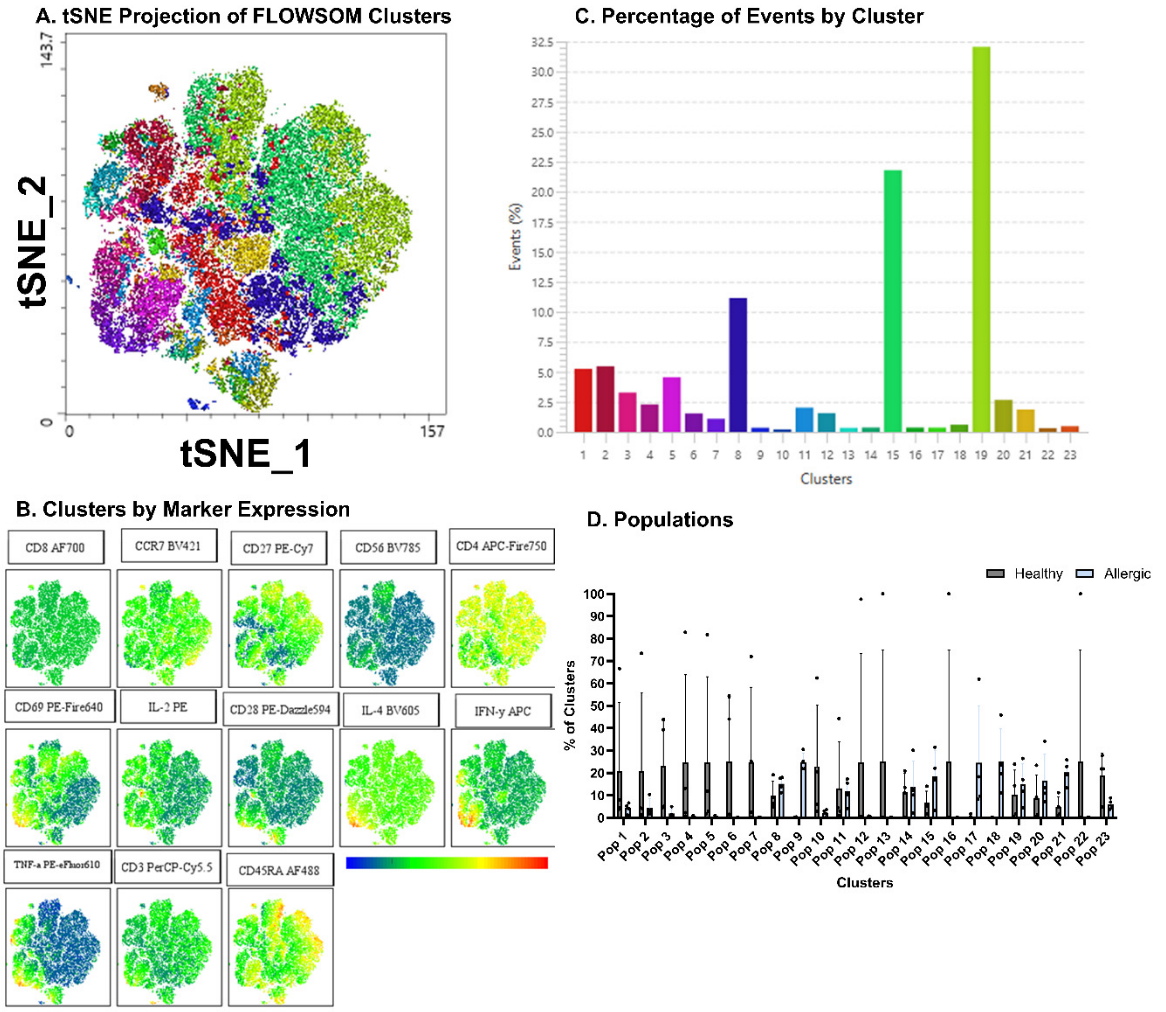

Supplementary Figure S4 - Comparison between healthy and allergic using unbiased clustering analysis following culture with unstimulated DC-derived EVs. Following cluster explorer plugin, a multi-coloured tNSNE plot was made (A) and individual marker clusters were produced from tSNE plot (B). Clusters were separated based on their % (C). (C). Populations between healthy and allergic groups were analysed for significant differences using Mann-Whitney Tests $p<0.05$ (D). N= 4 healthy donors, 4 allergic donors.

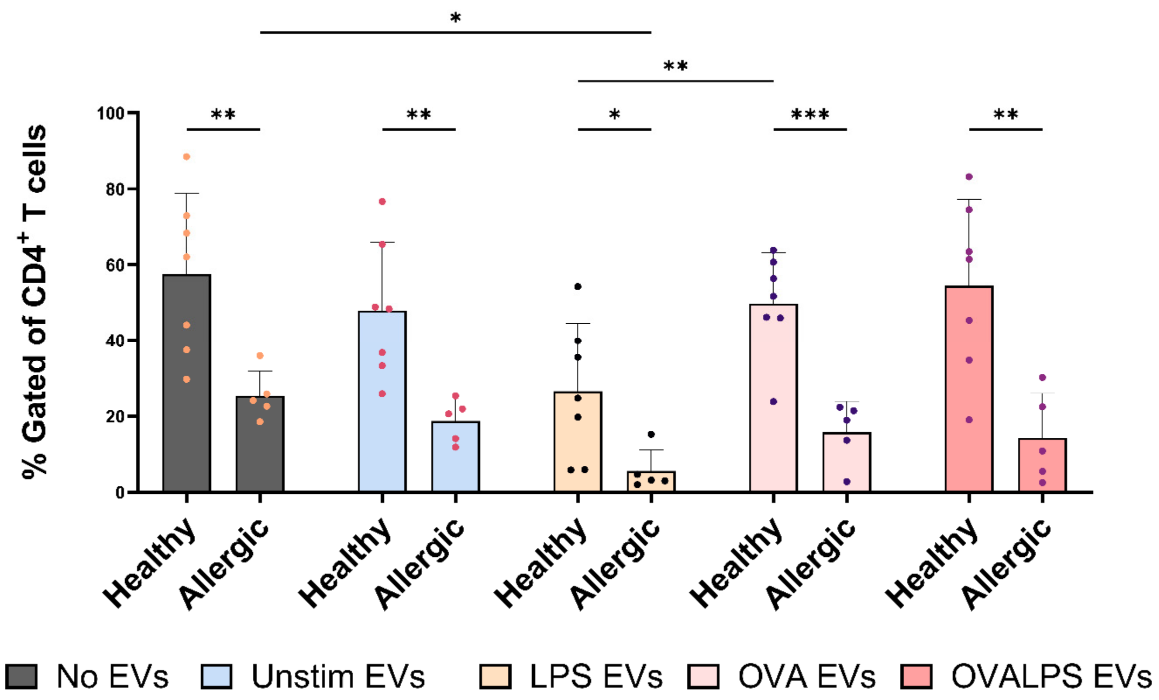

Supplementary Figure S5 - IFN-γ production by naïve CD4+ T cells in healthy and egg-allergic individuals. Naïve CD4+ T cells from healthy donors (n = 7) and egg-allergic donors (n = 5) were stimulated with plate-bound anti-CD3 and soluble anti-CD28 for 48 h with two doses of highly purified DC-derived EVs added at 0 h and 24 h. EVs were generated from monocyte-derived DCs that were either left unstimulated (UNSTIM EVs), stimulated with LPS alone (LPS EVs), OVA alone (OVA EVs), or OVA + LPS (OVALPS EVs). Cells were stained intracellularly for IFN-γ and analysed by spectral flow cytometry. Data show the percentage of IFN-γ+ cells among live CD4+ T cells and are presented as mean ± SD. Statistical significance was determined by two-way repeated-measures ANOVA followed by Tukey's multiple comparisons test (*$p < 0.05$, **$p < 0.01$, ***$p < 0.001$).
